# Krill oil extract inhibits the migration of human colorectal cancer cells and down-regulates EGFR signalling and PD-L1 expression

**DOI:** 10.1186/s12906-020-03160-7

**Published:** 2020-12-07

**Authors:** Abilasha G. Jayathilake, Margaret F. Veale, Rodney Brain Luwor, Kulmira Nurgali, Xiao Q. Su

**Affiliations:** 1 Institute for Health and Sport, Victoria University, P.O. Box 14428, Melbourne, Vic 8001 Australia; 2 Department of Surgery, The Royal Melbourne Hospital, The University of Melbourne, Parkville, Australia; 3 Department of Medicine, Western Health, The University of Melbourne, Melbourne, Australia; 4 Regenerative Medicine and Stem Cell Program, Australian Institute for Muscular Skeletal Science (AIMSS), Melbourne, Australia

**Keywords:** Krill oil extract, Human colorectal cancer cells, Migration, EGFR, PD-L1

## Abstract

**Background:**

The currently available treatments for colorectal cancer (CRC) are often associated with serious side-effects. Therefore, the development of a novel nutraceutical agent may provide an alternative complementary therapy for CRC. Overexpression of the epidermal growth factor receptor (EGFR) associates with a range of cancers while downregulation of EGFR signalling can inhibit cancer growth. Our previous studies have shown that the free fatty acid extract (FFAE) of krill oil exhibits anti-proliferative and pro-apoptotic properties. This study determines the effects of krill oil extract on the migration of human CRC cells, and its potential role in modulating EGFR signalling pathway and the expression of programmed death ligand 1 (PD-L1).

**Methods:**

Human CRC cells, DLD-1 and HT-29 were treated with FFAE of KO at 0.03 and 0.12 μL/100 μL for 8 or 24 h. Cell migration was determined by Boyden chamber migration assay. The expression of EGFR, phosphorylated EGFR (pEGFR), protein kinase B (AKT), phosphorylated AKT (pAKT), extracellular signal regulated kinase (ERK1/2), phosphorylated ERK1/2 (pERK1/2) as well as PD-L1 were assessed by western blotting and immunohistochemistry.

**Results:**

The FFAE of krill oil significantly inhibited cell migration compared to ethanol-treated (vehicle control) cells (*P* < 0.01 to *P* < 0.001). At the molecular level, krill oil extract reduced the expression of EGFR, pEGFR (*P* < 0.001 for both) and their downstream signalling, pERK1/2 and pAKT (*P* < 0.01 to *P* < 0.001) without altering total ERK 1/2 and AKT levels. In addition, the expression of PD-L1 was reduced by 67 to 72% (*P* < 0.001) following the treatment with krill oil extract.

**Conclusion:**

This study has demonstrated that krill oil may be a potential therapeutic/adjunctive agent for CRC attributed to its anti-migratory effects.. The potential anti-cancer properties of krill oil are likely to be associated with the downregulation of EGFR, pEGFR and their downstream pERK/ERK1/2 and pAKT/AKT signalling pathways along with the downregulation of PD-L1.

## Background

Colorectal cancer (CRC) is the third most common cancer in men and the second most common cancer in women worldwide [1, 2]. Estimated incidents of new cases exceed 1.8 million per year, causing 862,000 deaths globally each year [3]. The currently available therapies include surgery, chemotherapy, radiotherapy or a combination of them [4]. Complete surgical resection is curative only if the disease is identified at the early stage. However, initial stages of CRC are often asymptomatic and the percentage of stage IV diagnosis is only 20–25% [5]. Therefore, a high proportion of patients show distant metastases at the time of diagnosis [6]. The most common treatment for the patients with metastatic CRC is chemotherapy [7], and this is associated with serious side-effects [8]. Therefore, an alternative therapy with few or no adverse effects would be desirable.

Long-chain omega-3 polyunsaturated fatty acids (LC n-3 PUFA), eicosapentaenoic acid (EPA, 20:5n-3) and docosahexaenoic acid (DHA, 22:6n-3), commonly found in fish and other seafoods, have shown beneficial effects on several types of cancer including CRC [9]. The positive impacts of LC n-3 PUFA on CRC include inhibiting cell proliferation [10], metastasis and growth [11]. In addition, they improve patients’ immune function and reduce the toxicity and side-effects of chemotherapy [12]. Krill oil, extracted from small crustaceans *Euphausia surperba* from the Antarctic Ocean, is one of the rich sources of LC n-3 PUFA [13]. The LC n-3 PUFA in krill oil are bound to the phospholipids while in fish oil they are bound mainly to the triglycerides [13, 14]. It has been suggested that the bioavailability of phospholipid bound n-3 PUFA is higher than those bound to triglycerides and this may lead to more health benefits [15, 16]. Our previous studies have shown that the free fatty acid extract (FFAE) of krill oil inhibits the proliferation of both CRC and osteosarcoma cells, and induces the apoptosis of CRC cells [17, 18]. We also found that the anti-proliferative property of krill oil is comparable with a chemotherapeutic drug, Oxaliplatin [19]. Furthermore, we have reported that the anti-proliferative property of krill oil is associated with the activation of caspase-9 and caspase-3 leading to DNA damage in the CRC cells. Preliminary study by Zhu et al. also observed that krill oil treatment results in a time-dependent inhibition of CRC cell growth [20].

Epidermal growth factor receptor (EGFR) is a member of the erythroblastosis oncogene B (ErbB)/ family of receptor protein tyrosine kinase (TK) that transmits growth-inducing signals to cells [21]. The EGFR is stimulated by its interaction with the corresponding ligands. It then phosphorylates and activates several downstream signalling pathways including Ras/Raf/mitogen-activated extracellular signal-regulated kinase (Ras/Raf/MEK/ERK), phosphoinositide 3-kinase/ protein kinase B/ mammalian target of rapamycin (PI3K/AKT/mTOR). The overexpression of EGFR that correlates with cancer cell proliferation, tumour growth, invasion and metastasis is common in human cancers including CRC [22]. Therefore, the inhibition of EGFR signalling has been reported as an important target in cancer therapy [23]. Furthermore, it was found that the activation of EGFR and its downstream AKT signalling pathway is associated with an increased expression of the programmed death ligand 1 (PD-L1) protein [24, 25]. PD-L1, through its immune suppressive properties, plays multiple roles in several types of cancer such as, accelerating tumour progression, transmitting intracellular anti-apoptotic signals and improving cancer cell survival [26, 27]. The aims of this study were to investigate the effect of FFAE of krill oil on migration of human CRC cells; and determine the role of krill oil extract in modulation of EGFR and its downstream signalling pathways.. Furthermore, the efficacy of krill oil extract on PD-L1 expression was assessed.

## Methods

### Cell lines and culture conditions

The human colon adenocarcinoma cell lines, DLD-1 and HT-29 were obtained from the American Tissue Culture Collection (ATCC), Manassas, VA, USA (Catalogue No. CCL-221, HTB-38). Both cell lines were maintained in RPMI1640 medium (Sigma Aldrich, Castle Hill, NSW, Australia) supplemented with foetal calf serum (FCS, 10%) (Hyclone Quantum Scientific, Clayton South, VIC, Australia), glutamine (10 mM), 4–2-hydroxyethyl-1-piperazineethanesulfonic acid, sodium pyruvate (10 mM) and penicillin (100 U/mL)/ streptomycin (100 μg/mL) (Sigma Aldrich, Castle Hill, NSW, Australia). Cells were grown at 37 °C in 5% CO_2_ humidified atmosphere. Exponentially growing cells that were > 90% viable were used for assays.

### Extraction of free fatty acids from krill oil

Free fatty acids were extracted from the krill oil (Swisse Wellness Pty Ltd., Victoria, Australia) following the hydrolysis (saponification) method of Salimon et al. [28]. The extracts were dissolved in 100% ethanol and stored at -20 °C. The final treatment solutions contained < 0.1% ethanol as a solvent.

### Cell morphology assay

DLD-1 and HT-29 cells were seeded and cultured at 3 × 10^5^ cells per well in 6-well plates for 24 h. They were then treated with FFAE of krill oil for 24 h at two concentrations, 0.03 μL and 0.12 μL/100 μL (equating to the concentrations of EPA and DHA per 100 μL well at 0.13/0.06 and 0.52/0.26 μM, respectively). These two concentrations and duration of treatment were selected based on data from our preliminary experiments. All treatments were performed in triplicates and the results were verified through three individual experiments. In all experiments, 0.1% ethanol was used as a vehicle control. Cell morphology was analysed using the Olympus 1 × 81 (20X) microscope.

### Boyden chamber transwell migration assay

**Fig. 1:**
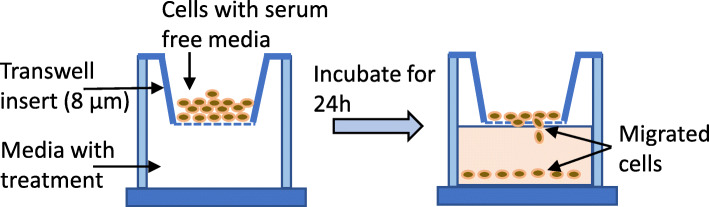
A schematic diagram of the transwell model

Cell culture was prepared in a 12-well Boyden chamber with filter membranes of 8.0 μm pore size (Corning, USA) to determine the cell migration. Cells were treated with serum-free medium for 24 h. A suspension of 1 × 10^5^ cells in 200 μL of serum-free RPMI 1640 media was added to the upper chamber of each well in the transwell plate. The bottom chambers were filled with 600 μL of serum free RPMI 1640 media supplemented with FFAE of krill oil at two dilutions of 0.03 μL/100 μL and 0.12 μL/100 μL. The control well (vehicle control) was filled with 10% FCS in culture media and less than 0.1% ethanol as a chemo attractant. The culture plates were incubated at standard culture conditions for 24 h. The wells and bottom of the transwells in a culture plate were then trypsinised (Fig. 1). The number of migrated cells were counted using a haemocytometer. All treatments were performed in triplicates and each experiment was repeated three times for both cell lines. The results were presented as mean ± SEM.

### Cell apoptosis

Flow cytometry was used to investigate the cell apoptosis by double staining with annexin V (Cat. 64,906 Bio Legend, Australia) and propidium iodide (PI) (Cat. P4864 Sigma-Aldrich, USA). Briefly, 3 × 10^5^ cell/well in 6-well plates were cultured for 24 h and then treated with FFAE of krill oil at the concentration of 0.12 μL/100 μL for 8 h. The cells were trypsinised and centrifuged at 1200 rpm at room temperature for 5 min and the cell pellets were resuspended in 100 μL of annexin V Binding Buffer (AVBB) (Australian Bio Search, Western Australia) containing 1 μL of PI and 1 μL of annexin V- FITC and incubated at room temperature in the dark for 15 min before 400 μL of AVBB was added. Cell apoptosis was detected using a FACS Canto II flow cytometer (BD BioSciences, Palo Alto, USA) and 10,000 cells were collected and analysed per sample. Control cells were treated with 0.1% ethanol. The data were analysed using the FACS Diva Software (BD Bioscience). PI was used to distinguish live and dead cells. All treatments were performed in duplicates and each experiment was repeated three times for both cell lines. The results were presented as mean ± SEM.

### Immunocytochemistry

Cells were seeded at a density of 2 × 10^4^ cells/well in 24 well plates with coverslips and incubated for 24 h. The cells were then treated with FFAE of krill oil at the concentration of 0.12 μL/100 μL for 8 h. Immunocytochemistry assay was performed as described in our previous study [19]. Target protein levels were observed using primary antibodies against pEGFR (1:500, rabbit, Cell Signalling, MA, USA), EGFR (1:500, rabbit, Cell Signalling), pERK1/2 (1:500, rabbit, mAB, 9101, Cell Signalling Technologies, MA, USA) and pAKT (1:500, rabbit, mAB, 9271, Cell Signalling Technologies, MA, USA). The cells were then incubated with secondary antibodies, Alexa Fluor 594 conjugated anti-rabbit or Alexa Fluor 488 conjugated anti-rabbit (Jackson Immuno Research Laboratories, PA, USA) (diluted 1:250) at room temperature for 2 h. Finally, cells were exposed to DAPI for 2 min and images were taken with the Eclipse Ti Confocal laser scanning system (Nikon, Tokyo, Japan). The excitation wavelengths for FITC and Alexa Fluor 594 were adjusted to 488 nm and 559 nm respectively. Each fluorophore was measured using 8 images taken at 20X magnification with a total area of 2 mm^2^. All images were then calibrated for a minimum basal fluorescence and converted into binary. Fluorescence intensity was measured using Image J software (National Institute of Health, USA). All treatments were performed in triplicates and the results were verified in at least three individual experiments.

### Western blotting

The expression of pEGFR/EGFR, pERK/ERK 1/2 and pAKT/AKT proteins was determined in DLD-1 and HT-29 cells treated with FFAE of krill oil at the concentrations of 0.03 μL and 0.12 μL/100 μL for 8 h and the results were compared with the ethanol vehicle control. Two additional steps were performed for pEGFR/EGFR expression: (i) cells were incubated with anti-EGF (Thermo Fisher Scientific) antibodies (1:1000 dilution with cell culture media) for 1 h before the FFAE of krill oil was added or cetuximab (Merck, Darmstadt, Germany) treatments, including the ethanol vehicle control; (ii) cetuximab (20 μg/mL) treated cells were used as a positive control. Western blotting was performed as described in our previous study [19]. Briefly, cell lysates were prepared and aliquots of each (12 μg) and separated using 4 to 20% sodium dodecyl sulphate (SDS)/polyacrylamide gel electrophoresis and the separated proteins were transferred to polyvinylidene fluoride (PVDF) membranes (Trans-Blot Turbo Transfer System, Bio Rad, USA). The membrane was incubated with primary antibodies rabbit anti-human against pEGFR (1:1000, Cell Signaling Technology, MA, USA), EGFR (1:1000, Cell Signaling Technology, MA, USA), pERK 1/ 2 (1:1000, rabbit, mAB, 9101, Cell Signalling Technologies, MA, USA), ERK 1/ 2 (1:1000, rabbit, H72, Cell Signalling Technologies, MA, USA), pAKT (1:1000, rabbit, mAB, 9271, Cell Signalling Technologies, MA, USA), AKT antibody (1:1000, rabbit, mAB, Cell Signaling Technology, MA, USA) and GAPDH as a control (1:2000 dilution, rabbit, Santa Cruz Biotechnology, USA) for overnight at 4 °C. Next the membrane was incubated with secondary antibody, goat anti-rabbit IgG H&L horseradish peroxidase (HRP) (Abcam, ab6721, MA, USA) at room temperature for 1 h. The protein detection was analysed using enhanced chemiluminescence reagents (ClarityTM Western ECL Substrate, Bio-Rad, USA) and visualised using a FUSION FX Densitometer (Vilber Lourmat, Germany). The expression level of each protein was quantified using Fusion Capt advance FX7 software. All treatments were performed in triplicate and the results were verified through at least three individual experiments.

### Statistical analysis

All data were analysed using SPSS 22 software (IBM, USA). Mixed model ANOVA was used to determine the significance between treatments. The significance of repeated measure at different time points was analysed using one-way ANOVA. Post-hoc analysis was conducted using Tukey HSD test for multiple comparisons. *P* < 0.05 was considered as significant. The results were expressed as mean ± SD in tables or mean ± SEM in figures.

## Results

### Effects of FFAE of krill oil on the viability, migration and apoptosis of CRC cells

**Fig. 2:**
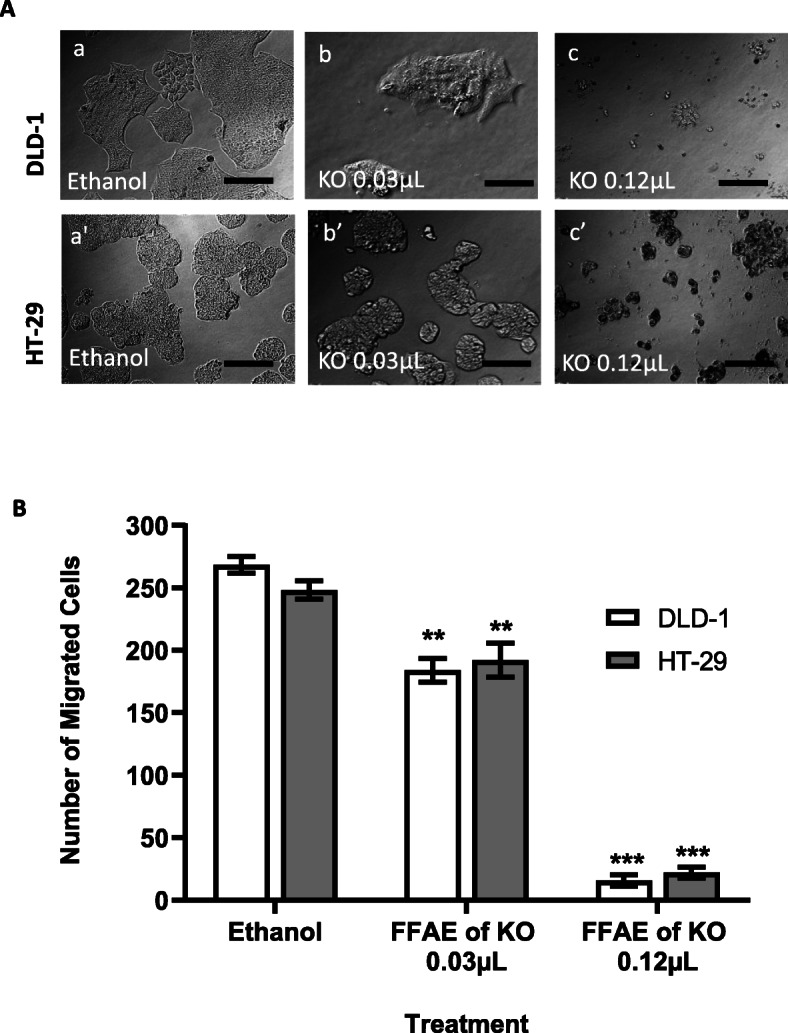
Effects of FFAE of krill oil on the viability of CRC cells

Morphological changes of colon cancer cells, DLD-1 and HT-29, treated with low and high doses of FFAE of krill oil, were analysed using fluorescent microscopy with a 20X objective (Olympus 1X-81, Nikon, Japan). As shown in Fig. 2a, the untreated cells displayed normal CRC morphology as indicated by their uniform distribution and confluence. However, the cells treated with FFAE of krill oil at 12 μL/100 μL have exhibited obvious morphological changes, including the loss of cell adhesion, membrane shrinkage, shape alteration as well as decreased cell numbers compared to the control group.

The effect of FFAE of krill oil on the migration of DLD-1 and HT-29 CRC cells was determined using a Boyden chamber transwell migration assay (Fig. 2b). The lower concentration of FFAE of krill oil (0.03 μL/100 μL) reduced the migration of DLD-1 cells by 31.4 ± 1.1% (*P* < 0.01) while the higher concentration (0.12 μL/100 μL) reduced the migration by 94.4 ± 1.0% (*P* < 0.001) following 24 h of krill oil extract treatment compared with the ethanol control. HT-29 cells treated with lower concentration of FFAE of krill oil at 0.03 μL/100 μL reduced the cell migration by 20.6 ± 1.1% (P < 0.01) while the higher concentration at 0.12 μL/100 μL resulted in 90.9 ± 2.3% reduction of HT-29 cell migration (*P* < 0.001) following 24 h of treatment.

**Fig. 3:**
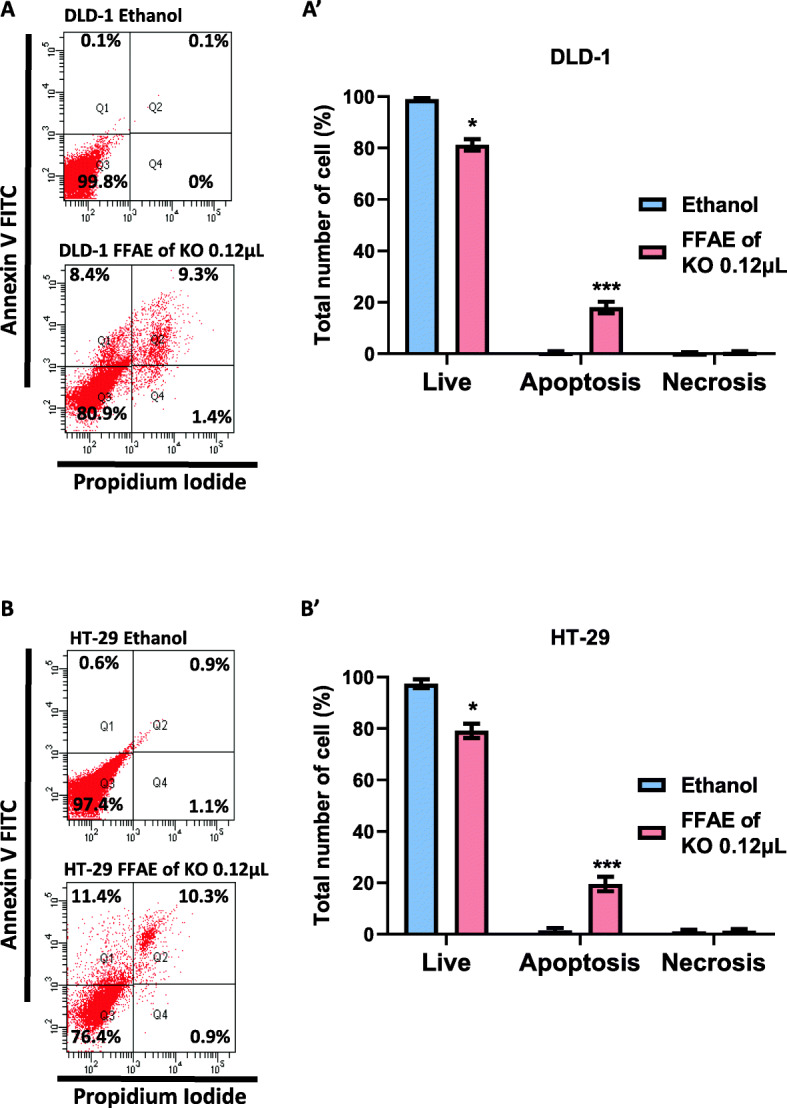
Quantification of apoptosis following the treatment with FFAE of krill oil

The effect of FFAE of krill oil on the apoptosis of two human CRC cell lines was investigated using an Annexin V/PI staining and flow cytometry (Fig. 3). The treatments with FFAE of krill oil at 0.12 μL/100 μL resulted in a significant reduction of viable cells (negative to both staining) and a significantly higher apoptosis rate across both CRC cell lines compared to the vehicle control cells. The percentage of apoptosis in DLD-1 and HT-29 cells has increased by approximately 18 to 19.5% following treatments with FFAE of krill oil for 8 h compared to ethanol-treated cells (Fig. 3A’ and B’). No significant cell necrosis was observed following treatments with krill oil in either cell line compared to ethanol-treated cells.

### Expression of pEGFR/EGFR, pERK/ERK 1/2, pAKT/AKT and PD-L1 following treatments with FFAE of krill oil

**Fig. 4:**
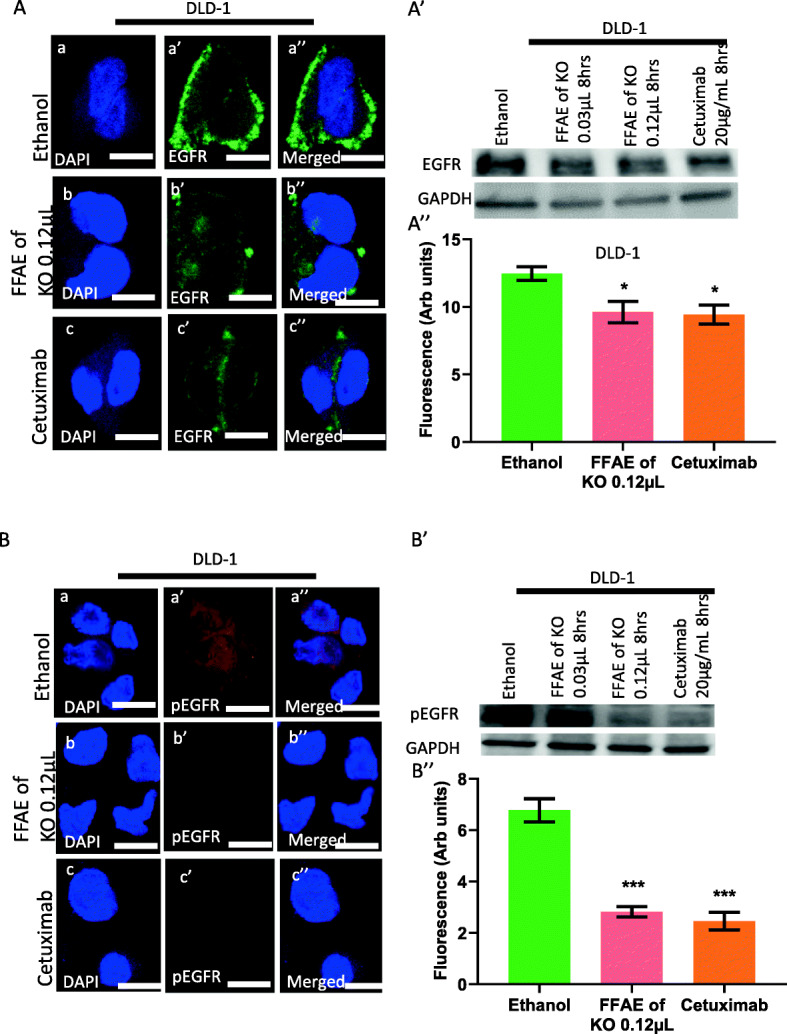
Expression of EGFR and pEGFR in DLD-1 cells following the treatment with FFAE of krill oil

**Fig. 5:**
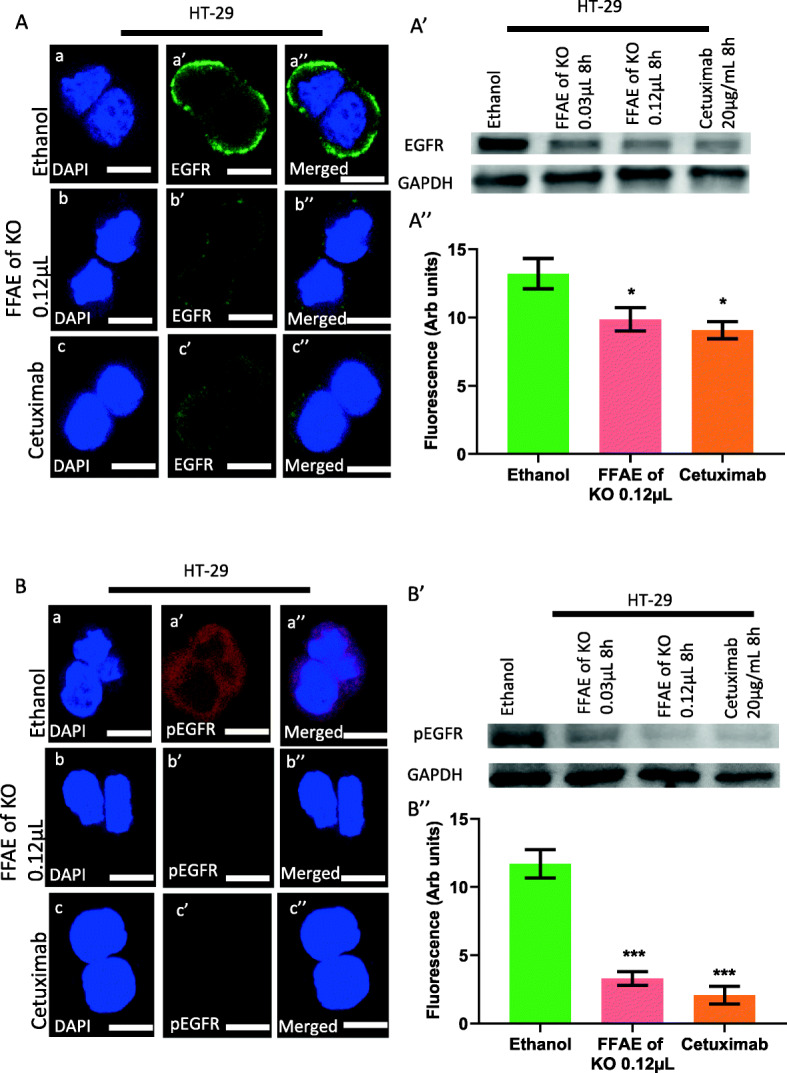
Expression of EGFR and pEGFR in HT-29 cells following the treatment with FFAE of krill oil

**Fig. 6:**
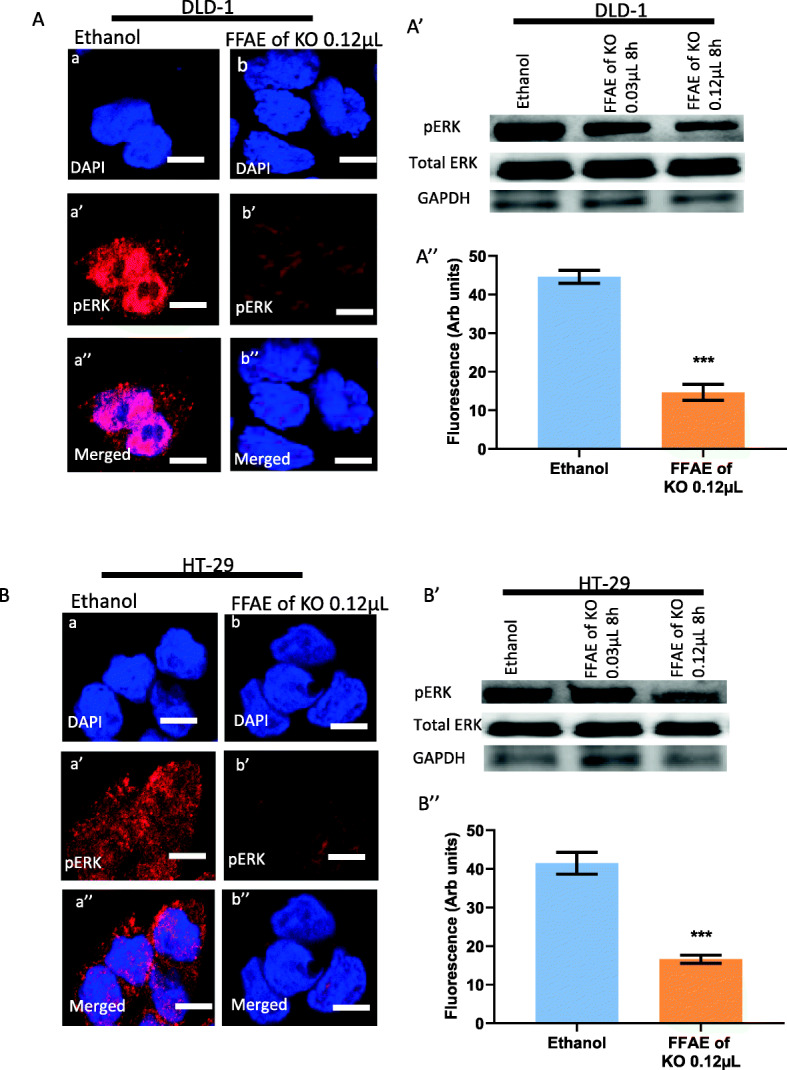
Expression of pERK 1/ 2 in CRC cells following the treatment with FFAE of krill oil

**Fig. 7:**
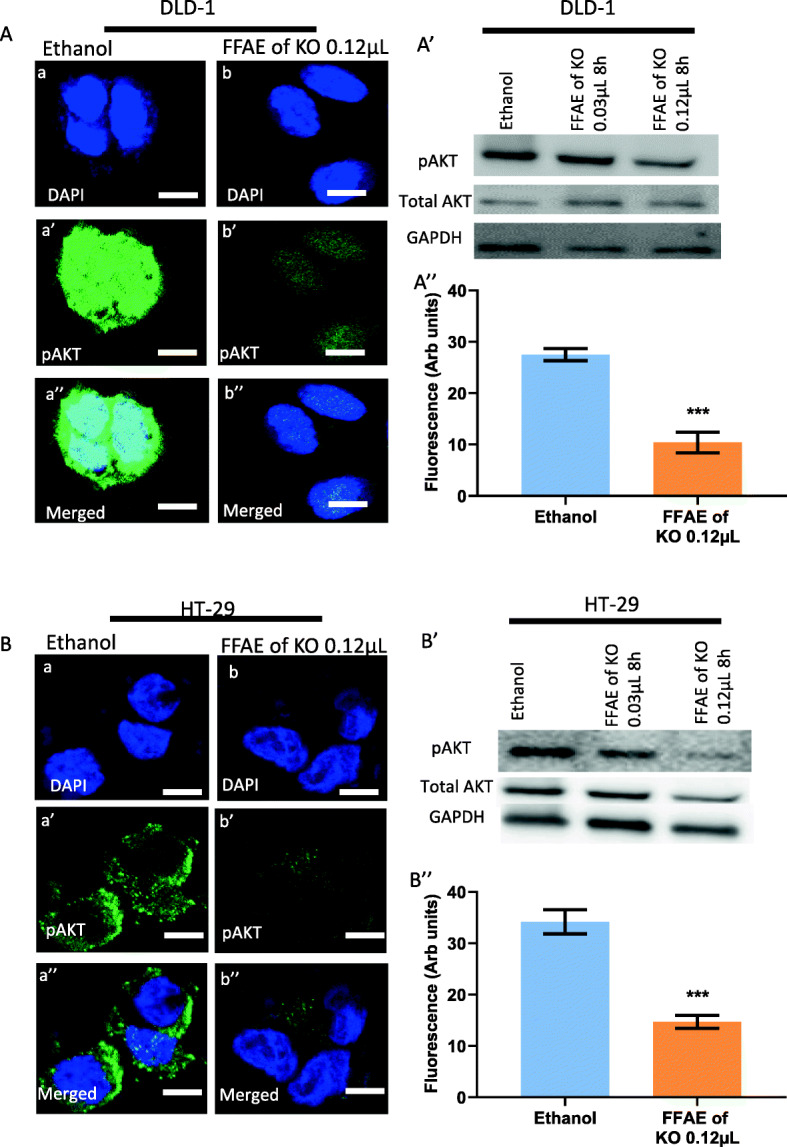
Expression of pAKT in CRC cells following the treatment with FFAE of krill oil

**Fig. 8:**
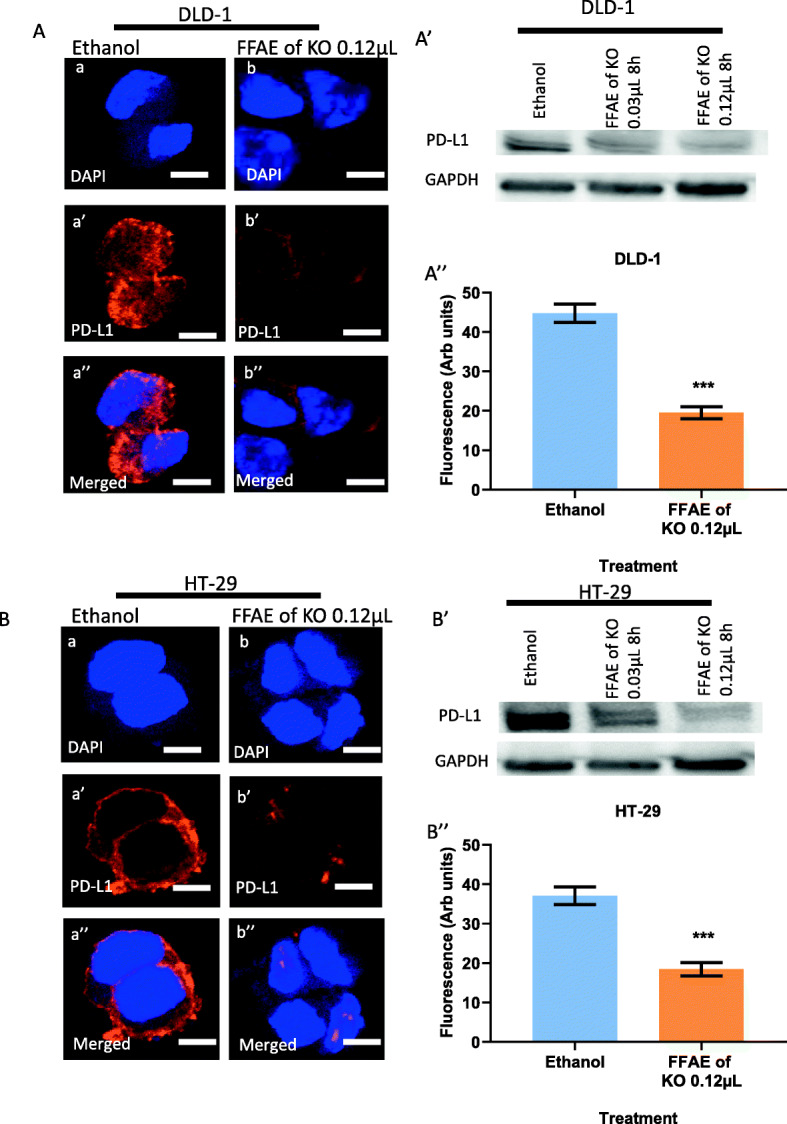
Expression of PDL-1 in CRC cells following the treatment with FFAE of krill oil

The expression of EGFR, pEGFR, pERK/ERK 1/ 2, pAKT/AKT and PD-L1 in DLD-1 and HT-29 cells was determined via western blotting and immunohistochemistry (Figs. 4, 5, 6, 7, and 8). The cells were treated at low (0.03 μL/100 μL) and high (0.12 μL/100 μL) concentrations of FFAE of krill oil for 8 h respectively before the proteins were extracted.

(A) Morphological changes of DLD-1 and HT-29 cells were observed using phase contrast microscopy at 20X magnification following 24 h of treatment with FFAE of krill oil compared with ethanol (vehicle control) treatment. Panel a, DLD-1 cells treated with ethanol; Panels b and c, DLD-1 cells treated with FFAE of krill oil at the concentrations of 0.03 μL/100 μL and 0.12 μL/100 μL respectively. Panel a’, HT-29 cells treated with ethanol; Panels b’ and c’, HT-29 cells treated with FFAE of krill oil at the concentrations of 0.03 μL/100 μL and 0.12 μL/100 μL respectively. Scale bar = 100 μM. (B) The number of migrated cells were quantified using the Boyden chamber assay. All treatments were performed in triplicate and repeated three times. Data are expressed as mean ± SEM. **p < 0.01 and ***p < 0.001 indicate a significant difference between the treatment of krill oil extract and ethanol (vehicle) control.

Apoptosis of DLD-1 (A) and HT-29 (B) cells were determined using Annexin V/PI staining and detected by the flow cytometry following 8 h of treatment with FFAE of krill oil at 0.12 μL/100 μL. Panels (A’) and (B’) show the percentage of live, apoptotic and necrotic cells compared with the ethanol vehicle control after treatments. All treatments were performed in duplicate and the experiment was repeated three times. Data are expressed as mean ± SEM. **p < 0.01 and ***p < 0.001 indicate a significant difference between the treatment of krill oil extract and ethanol (vehicle) control.

Figures 4 and 5 show the effects of krill oil extract and cetuximab treatments on the expression of EGFR and pEGFR after the stimulation by EGF. DLD-1 cells treated by FFAE of krill oil at the concentrations of 0.03 μL/100 μL and 0.12 μL/100 μL, and cetuximab at 20 μg/mL have shown a reduction in EGFR expression by 21.4, 27.9 and 29.9% respectively after treatment for 8 h compared to ethanol vehicle control (Fig. 4A’). A similar decrease in EGFR expression by 23.7, 31.8 and 29.9% was observed in HT-29 cells following treatments with FFAE of krill oil and cetuximab at the same concentrations for 8 h respectively compared to ethanol control (Fig. 5A’). Same treatments for DLD-1 cells have reduced pEGFR expression by 3.9, 49.0 and 56.6% respectively compared to the ethanol vehicle control (Fig. 4B’). Similarly, in HT-29 cells, reduced pEGFR levels by 36.7, 61.2 and 73.6% were also observed after 8 h of the same treatments compared to the ethanol vehicle control (Fig. 5B’). These data were further supported by the immunohistochemistry assay results shown in Figs. 4 and 5 (A”and B”). The FFAE of krill oil has effectively inhibited the levels of EGFR and pEGFR in both cell lines in a dose-dependent manner. The immunohistochemistry results are consistent with western blot results in both cell lines following the treatments with FFAE of krill oil at 0.12 μL/100 μL and cetuximab at 20 μg/mL for 8 h.

The expression of EGFR (A) and pEGFR (B) in DLD-1 cells was determined using confocal microscopy. The expression of EGFR (A’) and pEGFR (B’) in DLD-1 cells was measured by western blotting following 8 h of treatment with FFAE of krill oil at 0.03 μL/100 μL and 0.12 μL/100 μL, and Cetuximab (positive control) at 20 μg/mL. Fluorescent intensity of EGFR (A”) and pEGFR (B”) expression in DLD-1 cells was determined using a monoclonal antibody for EGFR and pEGFR following 8 h of treatment with FFAE of krill oil at 0.12 μL/100 μL and Cetuximab at 20 μg/mL. Scale bar = 50 μM. Magnification = 60X. The treatments were performed in triplicate and the results were verified through at least three individual experiments. Data are expressed as mean ± SEM. ***p < 0.001 compared to ethanol (vehicle) control.

The expression of EGFR (A) and pEGFR (B) in HT-29 cells was determined using confocal microscopy. The expression of EGFR (A’) and pEGFR (B’) in HT-29 cells was measured by western blotting following 8 h of treatment with FFAE of krill oil at 0.03 μL/100 μL, 0.12 μL/100 μL and Cetuximab at 20 μg/mL. Fluorescent intensity of EGFR (A”) and pEGFR (B”) expression in HT-29 cells was determined using a monoclonal antibody for EGFR and pEGFR following 8 h of treatment with FFAE of krill oil at 0.12 μL/100 μL and Cetuximab at 20 μg/mL. Scale bar = 50 μM. Magnification = 60X. The treatments were performed in triplicate and the results were verified through at least three individual experiments. Data are expressed as mean ± SEM. ***p < 0.001 compared to ethanol (vehicle) control.

The FFAE of krill oil also reduced the level of pERK1/2 and pAKT proteins without altering the total protein levels of ERK1/2 and AKT in both DLD-1 and HT-29 cells as shown in Figs. 6 and 7. DLD-1 cells treated by FFAE of krill oil at the concentrations of 0.03 μL/100 μL and 0.12 μL/100 μL for 8 h have shown a decrease in pERK 1/2 protein expression by 22 and 72% respectively compared to ethanol vehicle control (Fig. 6A’). A similar reduction of pERK1/2 protein level by 24 and 73% was also observed in HT-29 cells following treatments with FFAE of krill oil at the same concentrations for 8 h respectively compared to the ethanol vehicle control (Fig. 6B’). These results were further verified by immunohistochemistry assay findings (Figs. 6A” and B”).

The expression of pERK1/2 in DLD-1 (A) and HT-29 (B) cells was determined using confocal microscopy. The expression of pERK1/2 and total ERK1/2 in DLD-1 (A’) and HT-29 (B’) was measured by western blotting following 8 h of treatment with FFAE of krill oil at 0.03 μL/100 μL and 0.12 μL/100 μL. Fluorescent intensity of pERK1/2 expression in DLD-1 (A”) and HT-29 (B”) cells was determined using a monoclonal antibody for pERK1/2 following 8 h of treatment with FFAE of krill oil at 0.12 μL/100 μL. Scale bar = 50 μM. Magnification = 60X. The treatments were performed in triplicate and the results were verified through at least three individual experiments. Data are expressed as mean ± SEM. ***p < 0.001 compared to ethanol (vehicle) control.

DLD-1 cells treated by FFAE of krill oil at 0.03 μL/100 μL and 0.12 μL/100 μL have also shown a reduction of pAKT expression by 24.0, and 48.5% respectively after 8 h of treatment with FFAE of krill oil compared to ethanol control (Fig. 7A’). The results from similar treatments for HT-29 cells have shown a decrease of pAKT protein levels by 25.4 and 38.4% respectively after 8 h of treatment (Fig. 7A’). These results are consistent with the observations from immunohistochemistry assay (Figs.7A”and B”).

The expression of pAKT in DLD-1 (A) and HT-29 (B) cells was determined using confocal microscopy. The expression of pAKT and total AKT in DLD-1 (A’) and HT-29 (B’) cells was measured by western blotting following 8 h of treatment with FFAE of krill oil at 0.03 μL/100 μL and 0.12 μL/100 μL. Fluorescent intensity of pAKT expression in DLD-1 (A”) and HT-29 (B”) cells was determined using a monoclonal antibody for pAKT following 8 h of treatment with FFAE of krill oil at 0.12 μL/100 μL. Scale bar = 50 μM. Magnification = 60X. The treatments were performed in triplicate and the results were verified through at least three individual experiments. Data are expressed as mean ± SEM. ***p < 0.001 compared to ethanol (vehicle) control.

As shown in Fig. 8, the FFAE of krill oil has significantly reduced the PD-L1 expression in both DLD-1 and HT-29 cell lines after 8 h of the treatment. The DLD-1 cells treated with FFAE of krill oil at the concentrations of 0.03 μL/100 μL and 0.12 μL/100 μL showed a decrease in PD-L1 protein expression by 26 and 67% respectively after 8 h of the treatment compared to the ethanol vehicle control (Fig. 8A’). A similar effect was also observed in HT-29 cells with the reductions of 23 and 72% following 8-h treatment with FFAE of krill oil at concentrations of 0.03 μL/100 μL and 0.12 μL/100 μL respectively compared to the ethanol control (Fig. 8B’). The results of immunohistochemistry are consistent with the western blot results showing a significant decrease in the expression of PD-L1 in both DLD-1 and HT-29 cell lines compared to the ethanol vehicle control (*P* < 0.001) (Figs. 8A”and B”).

The expression of PD-L1 in DLD-1 (A) and HT-29 (B) cells was determined using confocal microscopy. The expression of PD-L1 in DLD-1 (A’) and HT-29 (B’) cells was measured by western blotting following 8 h of treatment with FFAE of krill oil at 0.03 μL/100 μL and 0.12 μL/100 μL. Fluorescent intensity of PD-L1 expression in DLD-1 (A”) and HT-29 (B”) cells was determined using a monoclonal antibody for PD-L1 following 8 h of treatment with FFAE of krill oil at 0.12 μL/100 μL. Scale bar = 50 μM. Magnification = 60X. The treatments were performed in triplicate and the results were verified through at least three individual experiments. Data are expressed as mean ± SEM. ***p < 0.001 compared to ethanol (vehicle) control.

## Discussion

This study demonstrated that the FFAE of krill oil inhibits the migration of human DLD-1 and HT-29 CRC cells. We also found that krill oil extract suppresses significantly the expression of EGFR, pEGFR and its downstream signalling, pERK1/2 and pAKT. In addition, the expression of PD-L1 was reduced remarkably following the treatment with krill oil extract. The validation analysis confirmed the pro-apoptotic properties of krill oil as we previously reported using other techniques and different CRC cell lines [18].

The positive impacts of krill oil extract observed in this study are more likely attributed to its bioactive constituents, LC n-3 PUFA, mainly EPA and DHA. Limited studies by our group and others have shown the inhibitory effects of FFAE of krill oil on the growth and proliferation of human osteosarcoma and CRC cells and those positive effects of krill oil were related to EPA and DHA [29, 30]. In addition, we have observed the anti-migratory effects of EPA and DHA on osteosarcoma cells [17]. Study by Zheng et al. has also found that DHA extracted from Antarctic krill inhibits the migration and invasion of breast cancer cells [31]. Previous in vitro and in vivo studies have reported the impacts of EPA and DHA, alone or in combination, on CRC suppression [32–34]. Several molecular mechanisms have also been proposed in relation to the role of LC n-3 PUFA in CRC inhibition including the modulation of cell membrane composition and structure [35], induction of anti-inflammatory response [12, 36], promotion of apoptosis via activation of caspases [37–39], as well as alteration of receptor binding and signal transduction process [37, 40]. A few studies have also shown the suppressive effects of LC n-3 PUFA on EGFR and the downstream signalling ERK and AKT pathways in CRC [41, 42]. However, there has been no report available on the effect of krill oil on EGFR signalling. Moreover, no data have shown the association of either krill oil or LC n-3 PUFA and PD-L1 expression.

EGFR signalling pathway is a complex and tightly controlled process in normal cells. Disruption of this system contributes to malignant transformation and formation of tumour through cell proliferation, prolonged survival, invasion and metastasis [22, 43]. The present study has demonstrated a significant decrease in the expression of both EGFR and pEGFR in CRC cells following the treatment with FFAE of krill oil. Furthermore, the suppressive effect of krill oil extract on EGFR and pEGFR is comparable with Cetuximab, a monoclonal antibody that binds to the EGFR extracellular domain and deactivates the EGFR receptor to inhibit tumour cell growth [44]. This suggests the potential clinical benefits of krill oil although further in vivo studies are warranted. The available data have shown that EPA and DHA play a role in modulating the EGFR signalling pathway in CRC cells. Turk et al. [42] have reported that EPA and DHA exert their anti-proliferative effect through the inhibition of EGFR in YAMC CRC cells associated with the alteration of membrane composition, fluidity and permeability. Changes in membrane property disturb the membrane raft formation resulting in the displacement of several raft-associated oncoproteins. It has been reported that the alterations in the lipid raft of cell membrane modify the localisation, suppression and activation of EGFR, as well as its downstream pathways in vitro and in vivo [32, 42]. Furthermore, a study on CRC HCT116 cells demonstrated that monoglyceride EPA (MAG-EPA) promotes apoptosis and inhibits tumour growth by suppression of the EGFR activation pathway [10]. A similar effect of LC n-3 PUFA has also been observed in MDA-MB-231 breast cancer cells. The alteration of lipid raft and reduction of EGFR level in these cells were found to be directly related to the inhibition of cell growth and the induction of cell apoptosis [45].

**Fig. 9:**
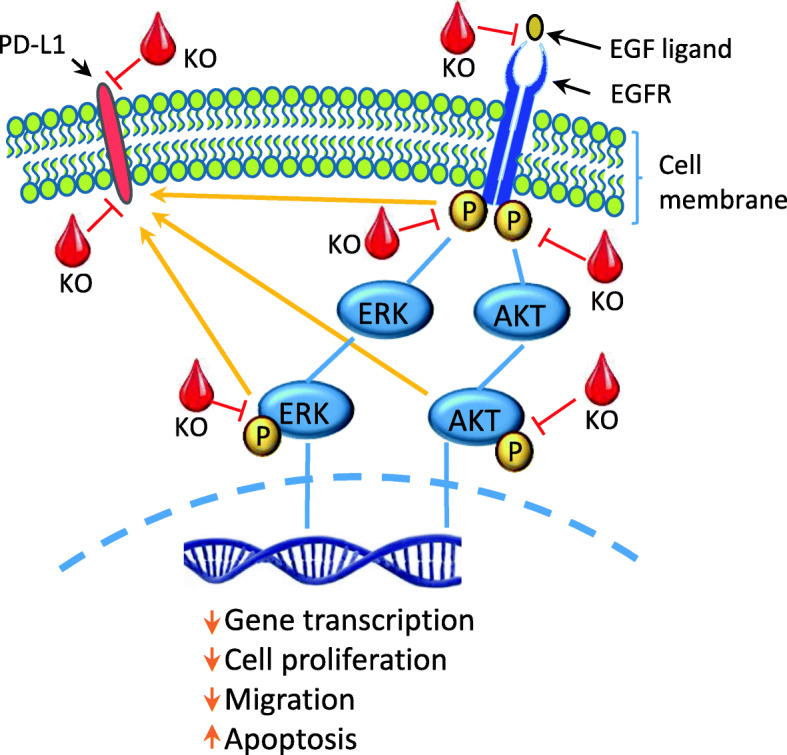
Schematic summary of the modulation of EGFR signalling pathway by the FFAE of krill oil in CRC cells

ERK1 and ERK2 are related protein-serine/threonine kinases involved in the Ras-Raf-MEK-ERK signal transduction cascade [46]. This cascade controls a large variety of processes including cell proliferation, differentiation, adhesion, migration, metabolism and transcription; and progression of cell cycle [23]. ERK1/2 is activated through the phosphorylation of tyrosine residues in ERK1 and ERK2 by phosphorylated MEK1/2 [46]. Phosphorylated ERK1/2 then translocates into the nucleus and regulates transcription factors leading to differential gene expression [47]. Several studies have shown that the activation of RAS/RAF/MEK/ERK pathways involves the promotion of CRC cell growth and proliferation [48]. The present study found that FFAE of krill oil reduces phosphorylated ERK1/2 expression in CRC cells in a dose-dependent manner. These results also correlate with the reduction of cell migration in both CRC cell lines tested in the study, suggesting the possible association between ERK signalling and anti-migratory property of krill oil extract (Fig. 9). Previous studies have also reported that EPA or DHA, alone or in combination, can reduce the phosphorylation of ERK1/2. Sun et al. [49] have observed that DHA treatment inhibits the phosphorylation of both MEK and ERK proteins and induces apoptosis in MCF-7 breast cancer cell line. Other studies have also reported that LC n-3 PUFA treatment decreases the phosphorylation of ERK1/2 in MIB-231 breast cancer cells [50]. Serini et al. [51] found that LC n-3 PUFA inhibits the phosphorylation of the MEK and ERK pathways.

The phosphoinositide 3-kinases (PI3Ks) are the family of lipid kinases that act as intermediate signalling molecules. These involve signal transduction from various growth factors and cytokines to induce the activation of multiple kinase pathways. One of the most well-known signalling pathways of PI3Ks is PI3K/AKT/mTOR [52, 53], and the phosphorylation of PI3Ks leads to the phosphorylation of AKT. The AKT signalling pathway plays a role in various normal cellular functions such as proliferation, growth, survival and migration. Furthermore, AKT is involved in protein synthesis, regulation of cell metabolism, DNA repair, apoptosis, angiogenesis and immune function [52, 54, 55]. It has been found that the dysfunction of PI3K/AKT signalling pathways is associated with the development of one-third of human cancers [52, 56, 57] and resistance to anti-cancer therapies [58]. The activation of AKT signalling in cancer cells promotes cell survival and proliferation, prevents apoptosis, stops DNA repair, activates pro-angiogenic target genes and cell metastasis [59].

Our results indicate that the level of pAKT in CRC cells was significantly reduced following the treatment with FFAE of krill oil. This was correlated with a decrease in cell migration and an increase in apoptosis thus suggesting that the anti-cancer potential of krill oil may be related to the downregulation of AKT signalling pathway. Previous studies showed that EPA and DHA decrease AKT expression, reduce cell proliferation and inhibit the growth of A459 non-small lung cancer cell line [60], PC3 and DU145 prostate cancer cells [61], human pancreatic ductal epithelial [26], HCT116 colon cancer [10] and MCF-7 human breast cancer cells [62]. Moreover, a dis-localisation of EGFR and alteration of AKT signalling pathway that initiates the pro-apoptotic process were observed in human breast cancer cell lines MCF-7, T47D and MDA-MB-231 following the treatment of EPA and DHA [50].

Programmed death ligand (PD-L1) is a cell surface protein of the B7 family and a specific ligand for the programmed death-1 (PD-1) receptors expressed in the activated T cells (CD4 + and CD8+) [63]. The high expression of PD-L1 is common in tumour cells. When PD-L1 binds to the corresponding receptor in T cells, it can strongly inhibit T cell activation and proliferation, and induces T cell apoptosis and then subsequently escapes immunosurveillance to promote further tumour growth [64–66]. Multiple signalling pathways regulate the PD-L1 expression. Some of the identified pathways are the hyper-activation of AKT-mTOR pathway [58, 67, 68] and the activation of ERK/MAPK pathways [67, 69, 70] resulting in the higher expression of PD-L1. Moreover, it has been reported that the activation of EGFR signalling pathway can up-regulate the expression of PD-L1 [71, 72]. The link between EGFR and activation of PD-L1 plays an important role in preventing cancer cells escaping the immune surveillance and, therefore the progression of cancer. This has been extensively studied in lung cancer cells and to some extent in lung cancer patients [73]. Limited studies on CRC cells showed that EGFR increases post-translational glycosylation and stability of PD-L1 [74]. It has been demonstrated that activation of EGFR mutation is associated with enhanced PD-L1 expression in human lung cancer cells which is reduced by EGFR inhibitors. Moreover, it was found that EGFR signalling regulates PD-L1 expression via AKT, p-ERK1/2/p-c-Jun, and MAPK pathways [70, 75–77]. Taken together, the present results suggest that krill oil extract inhibits EGFR and pEGFR expression and the downstream pERK/ERK1/2 and pAKT/AKT signalling which associates with reduction in PD-L1 expression. It implies that krill oil may have a potential in preventing cancer cells from escaping the immune surveillance. This interaction may have led to the inhibition of CRC cell migration (Fig. 9). Further investigation with EGFR knockout/knockdown cell lines is required to elucidate the mechanistic interplay between krill oil, EGFR and PD-L1.

The FFAE of krill oil inhibits the expression of EGFR and phosphorylated EGFR, its downstream signalling pathways of ERK1/2 and AKT, as well as PD-L1. These changes result in a suppressed proliferation, migration and promoted apoptosis of cancer cells. KO: free fatty acid extracted of krill oil.

## Conclusions

Our findings demonstrated that FFAE of krill oil reduces the migration of CRC cells. The health-benefitting effects of krill oil extract on CRC cells may be associated with the downregulation of EGFR and its downstream signalling pathways of ERK 1/2 and AKT. Furthermore, the FFAE of krill oil suppressed the expression of PD-L1 and thereby suggests it may have a potential in preventing cancer cells escape from immunosurveillance. These results indicate that krill oil may be a useful alternative complementary therapy for CRC. Further in vivo studies are required to validate these findings.

## Data Availability

The datasets from the present study are available from the corresponding author upon request.

## Abbreviations

CRC: Colorectal cancer
FFAE: Free fatty acid extract
EPA: Eicosapentaenoic acid
DHA: Docosahexaenoic acid
PUFA: Polyunsaturated fatty acids
LC n-3 PUFA: Long chain n-3 PUFA
FFAE of KO: Free fatty acids extractions of krill oil
EGFR: Epidermal growth factor receptor
pEGFR: Phosphoralated EGFR
PD-L1: Programmed death ligand-1
PI3K: Phosphoinositide 3-kinase
AKT: Protein kinase B
mTOR: Mammalian target of rapamycin
pAKT: Phosphoralated AKT
MAPK: Miotogen activated protein kinase
ERK: Extracellular signal regulated kinase
pERK: Phosphoralated ERK
STAT-3: Signal transducer and activator of transcription 3
PI: Propidium iodide
AVBB: Annexin V binding buffer
ATCC: American Tissue Culture Collection
FCS: Foetal calf serum
HEPES: 4–2- hydroxyethyl − 1-piperazineethanesulfonic
PBS: Phosphate buffered saline
PBS-T: Phosphate buffered saline + Tween 20
SD: Standard deviation
SEM: Standard error of mean
DAPI: 4,6-diamidino-2-phenylindole.
RIPA: Radioimmunoprecipitation assay buffer
SDS: Sodium dodecyl sulphate
GAPDH: Glyceraldehydes-3-phosphate de-hydrogenase
